# Supplementary material for: Circulating 20S proteasome for assessing protein energy wasting syndrome in hemodialysis patients
Source: PLoS One. 2020 Jul 31;15(7):e0236948. doi: 10.1371/journal.pone.0236948 (PMC7394422; doi:10.1371/journal.pone.0236948)
Supplement: Attachment — Submitted filename: Response to Reviewers.docx [file pone.0236948.s002.docx]

**Response to Reviewers**

**Editor comments**

1. We note that you have indicated that data from this study are available upon request. PLOS only allows data to be available upon request if there are legal or ethical restrictions on sharing data publicly. For information on unacceptable data access restrictions, please see http://journals.plos.org/plosone/s/data-availability#loc-unacceptable-data-access-restrictions.

In your revised cover letter, please address the following prompts:

a) If there are ethical or legal restrictions on sharing a de-identified data set, please explain them in detail (e.g., data contain potentially identifying or sensitive patient information) and who has imposed them (e.g., an ethics committee). Please also provide contact information for a data access committee, ethics committee, or other institutional body to which data requests may be sent.

b) If there are no restrictions, please upload the minimal anonymized data set necessary to replicate your study findings as either Supporting Information files or to a stable, public repository and provide us with the relevant URLs, DOIs, or accession numbers. Please see http://www.bmj.com/content/340/bmj.c181.long for guidelines on how to de-identify and prepare clinical data for publication. For a list of acceptable repositories, please see http://journals.plos.org/plosone/s/data-availability#loc-recommended-repositories.

We will update your Data Availability statement on your behalf to reflect the information you provide.

Reply : Because there are no restriction we have upload the minimal anonymized data set necessary to replicate our study finding as Supporting Information files

2. We note that you have included the phrase “data not shown” in your manuscript. Unfortunately, this does not meet our data sharing requirements. PLOS does not permit references to inaccessible data. We require that authors provide all relevant data within the paper, Supporting Information files, or in an acceptable, public repository. Please add a citation to support this phrase or upload the data that corresponds with these findings to a stable repository (such as Figshare or Dryad) and provide and URLs, DOIs, or accession numbers that may be used to access these data. Or, if the data are not a core part of the research being presented in your study, we ask that you remove the phrase that refers to these data.

Reply : Because the data are not a core part of the research being presented in our study we have remaved the phrases that refers to the data as requested (p11 l196 and p12 l216).

**Reviewer 1 comments**

In this manuscript Aniort et al, describes the role of C20s proteasome assay as a biomarker for the diagnosis of low muscle loss and PEW (protein energy wasting) in hemodialysis patients. For this purpose, they performed an ELISA to measure the 20S subunit in the blood of these patients but authors found out that C20s proteasome levels didn’t correlate with patients’ nutritional markers or clinical outcomes at baseline.

**Major flaws**

As authors already mentioned in the manuscript, the number of patients is too low.

For this reason, it is difficult to assess the statistic power of this analysis.

I would suggest to increase the number of cases and reperform the statistic.

Reply: We are aware that the number of patients analyzed is low. Unfortunately this clinical study has been completed to date and we cannot include new patients. Power to detect a correlation between two variables can be calculated. We used the triangular sequential test proposed by Rasch et al. Results were added in the discussion (p15 l285). The number of patients is too low for subgroups or multivariate analysis. However, we believe that we have sufficient statistical power (> 80%) to consider that circulating proteasome is not marker efficient enough to be usable in clinical practice.

**Minor flaws**

1. In Fig. 1 change “N” with “n” to maintain consistency.

This was corrected.

2. The work done by Aniort et al is of value but need further evidences. To improve the discussion, I want to suggest other ways to give an explanation on the data of ESRD subjects suffering from both diabetes and PEW. In this case the metabolic disorder of this court of patients might influence the levels of C20s proteasome subunit in some way unlike other patients affected by other comorbidity. In this perspective, an interesting point of view about this topic can be found in this paper (Niewczas M et al, Nat Med, 2019). The evidences contained in this work could be an input to wondering about new biomarkers that could correlate with PEW in a court of ESRD and diabetic patients at least.

Reply : We thank the reviewer for the comment. Indeed proteomic or transcriptomic are interesting ways to discover a panel of biomarkers predictive of muscle atrophy. We have added this reflection and the reference given as an example by reviewer 1 in the discussion part (p14 l274).

**Reviewer #2:**

The Aim of the study was to demonstrate that 20S proteasome assay can predict muscle mass loss and protein energy wasting in hemodialysis patients.

The study is informative and compelling. It provides a better understanding of chronic hemodialysis patients.

.
